# Expert consensus on best content of a robotic surgical curriculum: a systematic review

**DOI:** 10.1007/s11701-025-02893-2

**Published:** 2025-10-28

**Authors:** Anna K. Kieslich, Ruari Jardine, Hussain Ibrahim, Areeg Calvert, Kenneth G. Walker, Kim A. Walker, Angus J. M. Watson

**Affiliations:** 1 NHS Highland, Raigmore Hospital, Old Perth Road, Inverness, IV2 3UJ Scotland United Kingdom; 2 University of Aberdeen, Aberdeen, AB24 3FX Scotland United Kingdom; 3 Royal College of Surgeons of Edinburgh, Nicolson Street, Edinburgh, EH8 9DW Scotland United Kingdom

**Keywords:** Robotic assisted surgery, Curriculum, Surgical training, Delphi, Consensus, Simulation

## Abstract

Formal Robotic Assisted Surgery (RAS) training has not yet been introduced into surgical training in Europe. It is still unclear what exactly constitutes the appropriate content of a comprehensive robotic surgical curriculum. A systematic review of the evidence for RAS curricula and teaching was conducted on the platforms Medline, PubMed, Embase, CINAHL and PsycINFO using a Boolean combination of subject heading terms and keyword searches of robotic surgery with surgical training. After removal of duplicates 10,001 references were returned. Title, abstract and full text screening using the PICO framework for eligibility identified 175 relevant studies, out of which 28 studies focused on RAS expert opinion regarding the ideal curricular content. Data were extracted and analysed systematically using a predeveloped extraction tool, grouping results into curriculum elements, defined skills and curriculum content. Analysis focused on recurrent themes and differences among included studies. The review was registered on PROSPERO under ID Number CRD42024566778. The 28 studies (2008–2025) had a considerable degree of agreement on curriculum components, skills and implementation factors. The key components mentioned were e-learning, simulation, video, team training, non-technical skills (NTS), and modular operative training with proficiency-based progression to benchmarked metrics defined by surgical societies. RAS experts strongly recommend the implementation of a standardised RAS curriculum adaptable to surgeons’ skills and specialty with a common pathway. Simulation and objective assessment with feedback have overwhelming agreement among RAS surgeons. Intraoperative teaching should be modular, encompassing operative steps of increasing difficulty and defined by specialty societies.

**Supplementary Information:**

The online version contains supplementary material available at 10.1007/s11701-025-02893-2.

## Introduction

Intraoperative performance and surgical skill have been closely linked to patient outcomes. [1, 2] A notable variability, especially in minimally invasive surgery, has been demonstrated between individual surgeons’ skills and outcomes [3–5]. In an attempt to improve surgical outcomes there has been a considerable focus on errors, [6, 7] but optimisation of surgical training to improve outcomes has been under investigated globally. [8, 9]

RAS shares similarities with laparoscopic and open surgery but requires a unique technical and non-technical skill set, such as the machine ‘buttonology’, unique methods of retraction, situational awareness and communication [10–12]. An exponential uptake in robotic surgery, since its introduction in 2000[13], has not been followed by a formalised incorporation into the curricula for surgical trainees [14]. Currently industry provides most of the RAS training, which is more comprehensive than the simple device training [14] mandated by the United States Food and Drug Administration (FDA) on the introduction of robotic surgery [15]. Most of the robotic training in Europe has been provided for established surgeons. With an increasing availability of surgical robots in surgical departments, surgeons in training are now being involved in RAS, making a tailored curriculum necessary for the efficient use of training time.

**Fig. 1:**
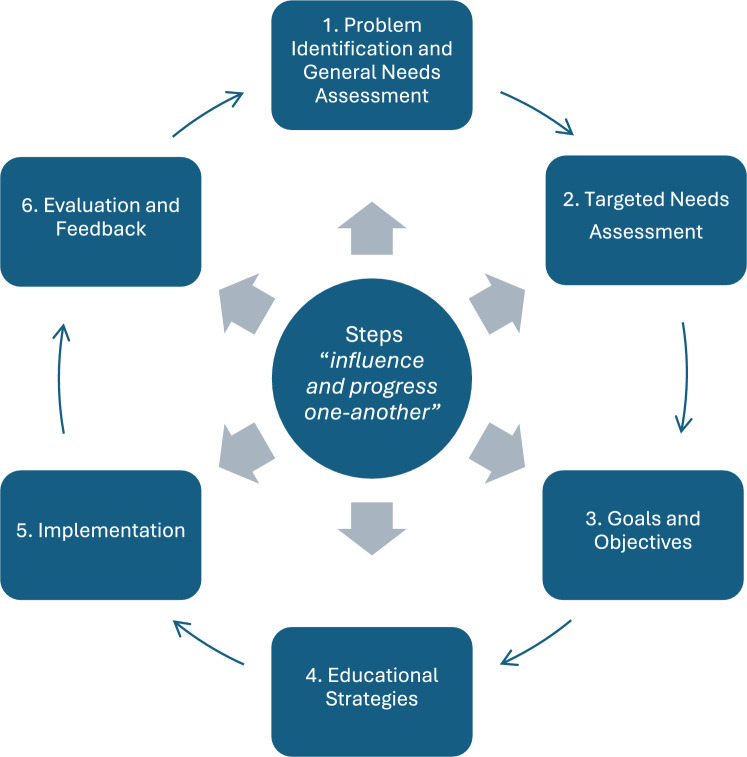
Kern’s Six-Step Approach to Curriculum Development Adapted from Kern et al.[17]

The main educational framework used for curricular development in the RAS literature is Kern’s 6 step approach [16]. Kern et al. proposed a ‘logical, systematic’ framework for healthcare curricula development and implementation, which incorporates both the healthcare need and the assessment of the curriculum itself emphasising the professional obligation of educators [17]. See Fig. 1

Ahead of RAS curricula development multiple program developers have performed consensus studies of RAS and educational experts to establish the needs assessment (steps 1 and 2) the learning objectives and benchmarks (step 3), and content of the curriculum (step 4). Currently, robust examinations of RAS curricula are still lacking and expert consensus, especially using the Delphi method, remains a useful approach to establish what the best RAS curricula content may be.

We aimed to systematically review RAS expert opinion on ideal RAS curricular content. This will inform part of a wider research question that is being addressed by a programme of work: what are the essential components of an effective robotic surgery training curriculum that are independent of specific robotic platforms, and what strategies can be employed for its successful implementation among trainee surgeons?

## Methods

### Search and sources

**Fig. 2:**
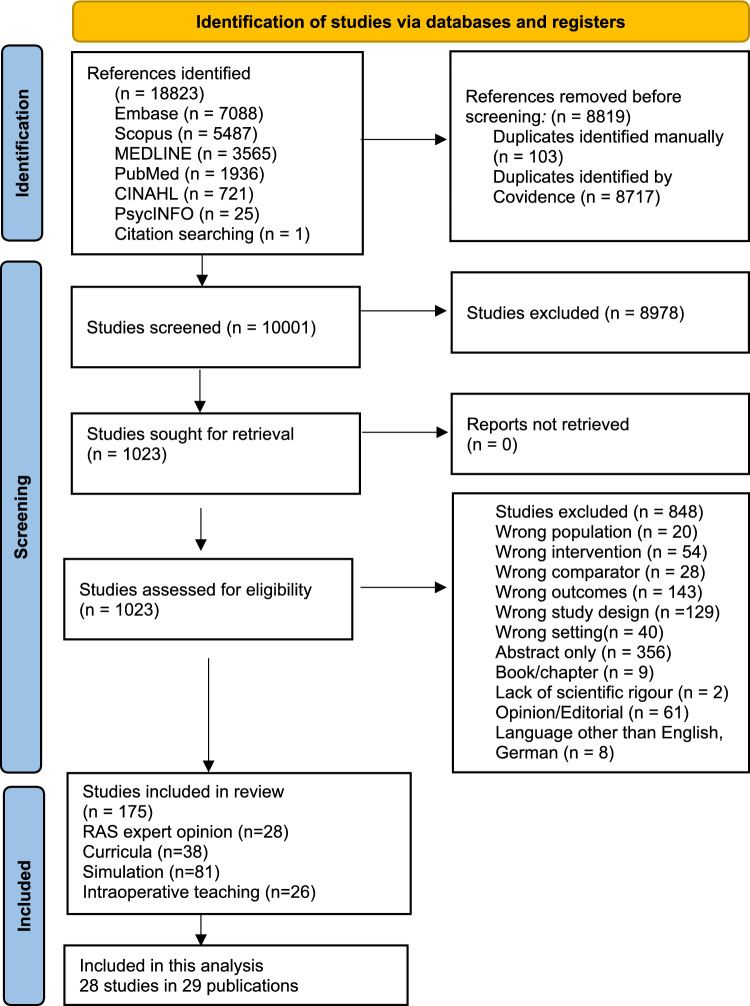
Flow chart of extraction process using PRISMA 2020 reporting methodology

This systematic review follows the PRISMA (Preferred Reporting Items for Systematic Review and Meta analysis 2020) Guidelines[18]. See Fig. 2 for PRISMA Flow Chart and Appendix 1 for PRISMA checklist. We used Covidence systematic review software ((Veritas Health Innovation, Melbourne, Australia, available at www.covidence.org) for title and abstract screening, full-text review and data extraction. Systematic platform-adapted Boolean literature searches, built with the aid of an information consultant and a librarian, combining RAS with training were conducted across six different databases (Medline, PubMed, Embase, Scopus, Cinahl, Psych Info) in English and German limited to the period from 1997, when the first RAS operations were performed, to current day (2025). Even though PubMed contains Medline a separate Medline search resulted in 512 additional articles, which we therefore included in the screening. See Appendix 1 for platform specific searches.

The searches were conducted between 17/01/2024 and 06/02/2024 after several scoping searches and updated on 20/06/2025. Reference chaining was used to ensure data saturation, and one additional reference was identified.

The search yielded 18,823 references, with10001 studies after exclusion of duplicates. Title and abstract screening excluded 8990 studies as irrelevant. Out of 1010 full-text studies assessed 175 studies met the inclusion criteria. 29 publications on 28 studies concerned expert opinion of RAS teaching. (PRISMA Flow-Chart Fig. 2) The review is registered on PROSPERO under ID Number CRD42024566778 on 02/10/2024.

### Study selection

We defined study eligibility criteria using the PICO (population, intervention, comparator, outcomes) framework (Table 1).

**Table 1:** PICO inclusion/exclusion criteria

Eligibility Framework	Inclusion	Exclusion
Population	Surgeons	Medical students, other health professionals
Intervention	Curriculum and teaching elements	Questionnaires, ultrashort interventions (single trial on simulator/ one day course)
Comparator	Curricula, training methodology	Learning curve
Outcomes	Completion/ engagement in curriculum, meeting predefined standard, patient outcomes	Outcomes other than teaching/ training of robotic surgery e.g. simulator validation, feasibility of teaching, quality assessment mechanisms (crowd-based video review and robotic surgery assessment scores)
Setting	General Surgery, Urology, Gynaecology, cardiothoracic surgery	Orthopaedic surgery, Ear Nose and Throat (ENT) Surgery, Neurosurgery
Publications	Peer-reviewed original publications	Abstracts, Books, Literature Reviews, Questionnaires, Editorials, single surgeon case series

We focused the setting on RAS in the specialties of general surgery, gynaecology, urology and cardiothoracic surgery. These specialties have a comparable workspace in a confined cavity (compared to orthopaedic surgery and ENT Surgery) and use the Da Vinci platform (Intuitive, Sunnyvale CA, USA), as well as other emerging surgical robots such as the Versius (CMR Cambridge, UK) and the Hugo (Medtronic, Minneapolis MN, USA).

Although initially included in the search, we also excluded articles on learning curve as they were often presented as a case series and mostly did not describe the method of learning or teaching. AK plus one of RJ, HI, AC or AW did the title and abstract screening. AK plus one of RJ or AW did the full text review. Conflicts were resolved by discussion. Data extraction and analysis was done by one reviewer (AK) and cross-checked by another (AW) to reduce bias.

### Data extraction and analysis

The papers included in the systematic review were heterogenous. We analysed studies with comparable methodology and outcomes together. Here we analysed studies concerning RAS expert opinion on RAS training. Data were extracted systematically, using a previously developed extraction tool adapted to study methodology.

To assess the quality of the consensus studies, we extracted method / rounds / level of consensus, identification of themes voted on, and the number and type of participants.

To identify and describe the optimal RAS curricular format, curricular elements were grouped into curricular content and skills required. Both aspects were subdivided into knowledge, technical skills (psychomotor skills, intraoperative skills) and non-technical skills training. Additionally, the specific teaching requirements named by studies were extracted. These were grouped into: curriculum, trainer/training centre, industry, duration/exposure and assessment. Results can be grouped according to Kern’s curriculum development approach. Data were compiled in Covidence (Melbourne, Australia) extraction sheets and exported to Excel. Further analysis of word- frequency was aided by Lumivero (2023) NVivo (Version 14), (available at www.lumivero.com).

## Results

Our systematic review returned 176 publications. Out analysis was grouped by methodology and topic of the publications included. 28 studies (out of 29 publications) focused on expert opinion on RAS curricula content and teaching.. The studies from 2008–2025 were comprised of consensus documents (n = 25), and qualitative studies establishing RAS expert opinion (n = 4). For consensus studies the Delphi methodology (n = 18) and other methods of consensus (n = 7) were used. The studies included the following surgical specialties: general surgery (n = 12), urology (n = 5), gynaecology (n = 2), thoracic surgery (n = 3) and multispecialty (n = 8) [19–41]. Just over half of the studies mentioned the presence of experts in education as part of the consensus group. [21–23, 25–27, 29, 31, 32, 37, 39, 40, 42, 43]

As this analysis includes expert opinion only, the overall quality of evidence is low [44]. Not all studies specified the methodology of consensus. Additionally, the methodology used in the Delphi studies varied and many of them did not report the methodological details required for a recently published Delphi assessment tool [45]. Some authors participated in several studies. [19, 22, 24–27, 29, 30, 32, 33, 36, 38, 41, 43] Medtronic, Minneapolis MN, USA and Intuitive Surgical, Sunnyvale CA, USA funded several studies and authors. [22, 24–26, 29, 39, 46]This may have reduced the quality information further. [45]

None of the studies reported on stability of consensus across rounds of voting. One study reported the Disagreement Index (DI) (based on RAND method; with 0 being complete agreement and > 1significant disagreement or lack of consensus). [47]Panellist anonymity in the Delphi process was not specifically mentioned in several studies. [22–24, 26, 29, 32, 36–39, 43, 48] However, the use of an electronic method of consensus may imply anonymity [22–24, 27–29, 33, 36–38, 43, 46–50]. We have summarised the details of the study methodologies in Table 2.

**Table 2:** Summary of methodology and curricular elements of included studies

Study	Target/aim	Generation of themes/method of consensus	No/type of participants	Methods/% of consensus	Curricular components	Skills required technical and non-technical (NTS)	Educational theory and requirement	Assessment and certification
Herron et al SAGES 2008[19]	Gen. surg All surgeons; Consensus use of RAS, training/ credentialing	Lit. review, participants; 2-day Consensus conference	20; International multi-disciplinary consensus group	Method and % consensus not specified	E-learning, Video Simulation (VR^a^, DRY^b^, Cadaver) adequate OR volume	Device function/ limitation, patient selection, clarity of dissection, completing procedure NTS: Technology and team interaction, troubleshooting	Evaluation by standard criteria of competence/ Instructors: substantial practical experience Mentoring: unbiased, confidential, objective	Institution certifies, summary letter preceptor, ongoing review of recognised benchmarks
Smith et al FRS^c^ 2014[32]	All specialties all surgeons; Develop pre- procedure curriculum	Review of Dulan 2012 curriculum; 2 conferences, 3 workshops Delphi	1st 20; 2nd 38; surgeons, scientists, medical educators, behavioural psychologists psychometrician, facilitators, reps of governing and certifying organizations	3 conferences with Delphi after + workshop/ 80%	Lecture, Video, FRS Simulation (VR, Dry Lab), team training, WHO checklist	Energy, clutch, camera, Instrument exchange, Foreign Body management, Multi-arm control, Eye–hand instrument coordination, Wrist articulation, tissue handling, dissection – Needle driving, Suture handling, Knot tying, Safety of field NTS: Team crisis response	“Pbp”^d^ (competency-based progression), standards, metrics, errors	Scoring by visual observation on pre- defined criteria and errors
ERUS^e^ Ahmed et al 2015[25]	Urology All surgeons; Develop and implement RAS curriculum	Participants; Analysis of discussions, questionnaire 2013 ERUS attendees	39; RAS surgeons, engineers, medical educators	1Delphi round/ 80%	EUA online module, 5-day course (VR, DRY, WET^f^ Lab) 6 months OR^g^ fellowship incl. bedside assist, dual console	History of RAS, port placement, troubleshooting, camera, diathermy, safe tissue dissection, tying knots, suturing, transfer, dissection, correct wrist articulation in manoeuvring the robotic arms procedure specific operative modules (e.g. RARP^h^) NTS: Decision making, situation awareness, teamwork, communication	Pbp, modular training, dual console/ Nationally certified training centres	2 examiner anonymous video review
Petz et al CRSA 2016[20]	Colorectal, All surgeons; Introduction of RAS, design colorectal curriculum	Participants; -Round table -Nominal group technique	Not specified number of Experts	Equal opportunity to speak/ % consensus not specified	Cognitive teaching, Video, Simulation (VR, *cadaver only if available*), dual console	Device functioning, docking, port placement, arm collision avoidance, pedal coordination, camera control, 3rd arm use, tissue handling, 2 hand coordination, endowrist technology, knot-tying, suturing, energy devices, vessel dissection, bowel resection, intracorporeal anastomosis, pelvic dissection NTS: Decision making, NOTS are expected with surgical training	“Pbp”, “modular”^i^, practical tasks with immediate feedback/ Course directors and surgical proctors: -academic position -aptitude for training	Assessment specific to deconstructed operative steps, Institution certifies
Veronesi et al ESTS, EACT^j^ 2018[29]	Thoracic surgery, all surgeons; Develop Thoracic curriculum	Lit. review; Delphi	14 expert RAS Thoracic surgeons	3 Delphi rounds + in person discussion of borderline/ 80%	FRS, E- learning, Simulation (VR, DRY lab), *cadaver no benefit*, Video review, Min of 3 cases proctored	Trouble shooting, docking, port, patient selection and preparation, other work by group:25 specific RAS skills defined (incl needle driving, handling, energy sources, docking, system errors), patient turnaround, emergency scenario, decision making NTS: Team training, patient turnaround, emergency scenario, decision making	“Modular training”, “Pbp” training, success metrics/ Proctorship by certified thoracic surgeons with vast experience and motivation for teaching training centres accredited by society for volume and expertise of trainer	Video assessment 2 external examiner, continued outcome reporting NOTS assessment
Rusch et al SERGS^k^ 2019[46]	Gynaecology, All surgeons; European Gyn curriculum	Lit. review, ERUS curriculum; Delphi	12 Experienced surgeons and members of SERGS	3 Delphi rounds + discussion of results in panel/ 80%	FRS, E-learning, Video, Simulation (VR, DRY, WET) team training, min 10 cases operative training	Patient selection, port placement, docking, console psychomotor skills not defined NTS: Details of NOTS failed, but included consensus/ trouble shooting, team efficiency	Modular, “Pbp” Validated scoring system for consistent feedback/ Trainers, proctors, training centres (volume dependent) assessed and certified	Video assessment 2 external examiner
Larcher et. al ERUS 2019[26]	Urology, RAS surgeons attempting Nephrectomy; Structured curriculum for RAPN^l^	Lit. Review; Delphi	27 RAPN Experts	3 Delphi rounds- not described in detail/ 80%	E- learning, case observation, Simulation (VR, DRY, WET), operative praxis e.g. 18months fellowship	See VR RARP curriculum Dry Lab: Basic robotic dexterity, Suturing, Bulldog (hilar clamps) management Wet lab: living porcine model; Case Simulation Vascular injury; OR Modules I-V NTS: Decision making, emergency scenarios	Pbp, Modular training/ Host centre min. annual volume of 40 RAPN	Video assessment 2 external examiner
Vanlander et al ORSI/ OCERT^m^ 2020[33]	All specialties, all surgeons; standardised training methodology for pre- clin. course	Lit. review; Delphi	36 RAS surgeons, educationalists, 10 industry reps (not voting)	2 Delphi rounds, voting after 2 consensus conferences/ 80%	E-learning, technology/device training, 1 week specialty bootcamp	Skills not defined Specialty societies should design a specialty-/ procedure specific training course, using a consistent template NTS: No mention	Pbp, modular training (not intraoperative), validated metrics, deliberate practice, benchmarked training/ -Mentors evaluated by objective criteria - specialised simulation centre -courses approved to standard	Pbp Video assessment external examiner
Ismail et al BIARGS^n^ 2020[28]	Gynaecology trainees; Develop list of competencies, for core Gyn RAS curriculum	Participants; Delphi	14, 38 (round 3) Gynaecologic RAS surgeons/starting RAS program	4 Delphi rounds + national survey / 80%	E-learning, simulation, case observation, min. 15 cases supervised operative training	Room set-up, Safe entry/port placement, docking, clear image, instrument exchange, adjust arm position, clutching, multi-arm control, hand-eye, blunt/ micro dissections, safe cutting, needle driving, suturing, knot tying NTS: No mention	Competency based, procedural teaching, Pbp/	Assessment method not specified, continued outcome reporting min 25 cases/year
Fong et al HPB^o^ 2020[22]	HPB surgeons; Define and optimize RAS HPB training curriculum	Lit. review; Delphi	12 HPB Experts/ publication in training and education	3 Delphi rounds + document acceptance, 2 meetings in person 80%	FRS, Video, simulation (VR, DRY, WET, team), case observation by mentor, Video analysis with feedback	Hardware description, patient selection, port placements, docking, FRS, team training and efficiency, emergencies, operative skills not defined NTS: Emergency management, Situation awareness, communication, team training mentioned	“Pbp”, Modular training, Video analysis with feedback, machine-based metrics (Sim)/ trainers and proctors Certified, training centre accredited related to case volume	Video assessment 2 external examiner + Log-Book, continued outcome reporting
Levy et al FTRS^p^ 2021[30]	Thoracic surgeons; Standard Lobectomy curriculum	Lobectomy task deconstruction, Consensus conference	Not specified number of expert thoracic surgeons	Not specified	FRS, Video, simulation (VR, DRY, WET), team training	Module 1–5, 1: introduction, 2: pre-procedure patient assessment and preparation 3: bedside assistant 4: console surgeon NTS:5: team training	“Modular”, training items with defined errors, Deliberate practice, metrics	No mention
Schmiederer et al 2021[23]	General surgery trainees; Define Key factors of RAS intra-op independence	Lit. review, Participants, Delphi	29 RAS faculty and trainees (approx. half each)	2 Delphi rounds / 75%	Current training pathway: Intuitive online modules, in-service training, Simulation (Dry lab), bedside assist, in theatre console training, resident interest, proficiency in lap, industry certification	Aspects identified, which affect resident autonomy on the RAS console: Dual console model Robotic-Focused Faculty Resident Interest in Robotic Surgery Chief Resident (PGY^q^4-5) Resident Time Spent on Simulator NTS: no mention	Deliberate practice, Dual console	Certificate of da Vinci System Training currently possible, but not required
Stefanidis et al 2022[36]	All specialty, all surgeons; define RAS credentialing criteria	Lit. review, 43 US institutions Evaluated; Delphi	45/ 28 opinion leader RAS surgeons	3 Delphi rounds/ 80%	FRS (proposed by same group), Video review, Simulation with objective metrics /feedback, device and specialty specific	Not defined NTS: No mention	“Pbp”, deliberate practice, standard proficiency metrics/ benchmarks/ proctor ≠ preceptor, independence from industry, specialty specific	Preceptor, proctored, 2 examiner independent video review follow up, instrument metrics, Institution certifies
Fuchs et al RAMIE^r^ 2022[24]	Oesophageal surgeons converting; RAMIE curriculum	Lit. review; Delphi, virtual meeting after 3 rounds	14 International RAMIE opinion leaders	3 Delphi rounds/ 80%	E-learning, Video, device training, Simulation (WET), case observation FRS no consensus	Robotic hardware, patient selection/ preparation, trocar placement (basic and RAMIE specific), docking, trouble shooting, OR team efficiency, emergency undocking scenarios; instrument movement, pick and place, suturing, knot tying NTS: Emergency scenarios, Team training Situation awareness failed with 71%	Modular console training, “Pbp”, Video of performance with feedback, objective performance metrics, benchmarked proficiency/ trainers by caseload, high motivation to teach, caseload dependent centre accreditation	Video assessment 2 external examiner, re-evaluation audit
Hertz et al 2022[37]	All specialty, all surgeons; Common basic RAS curriculum	Participants; Delphi	56 RAS surgeons Gyn, Uro, Gen. surg. in Denmark	3Delphi rounds/ 75%	Classroom, E-learning, Self-study (books, online), simulation (VR, DRY, WET, Cadavers), team training, supervised surgery	Robotic platform/equipment/set-up, Port placement, Docking, arm control, Unforeseen events, Emergency situations, Patient positioning, camera control, dissecting, tissue handling, suturing, knot tying, haemostasis, understand absence of haptic feedback NTS: Team training, communication, troubleshooting, analyse errors	No mention	No mention
Dell’Oglio et al ERUS 2022[27]	Urology, RAS surgeons adopting Cystectomy, Structured RARC curriculum	Lit. review presumed; Delphi	28 Experts	1 Delphi round/ 80%	E-learning, case observation, 5Day pre-clinical course, simulation (VR, DRY, WET, Cadaver), NOTS simulation, operative practice, dual console	RARC case divided into 11 steps, grouped into V modules by complexity (from 1 easy to 5 complex) NTS: Simulation- Decision making Emergency scenario Team training, closed loop- communication	Modular, PbP, RARC specific scoring, benchmark metrics/ appropriate experience and skills, mentors trained to share experience, ERUS Host centre certification, 30 annual RARC	Video 2 external examiner
Burke et al ASiT^s^ 2023[38]	All specialty, Trainees; pre-procedural core robotic surgery curriculum (PPCRC)	Lit. review; Delphi	43 surgical trainees, ASiT representative, from med student to post CCT^t^	3 Delphi rounds / 80%	E-learning, simulation (VR, DRY), device training	Patient selection, preparation, trouble shooting, other skills not defined NTS: Situation awareness, team training, communication	Pbp, objective metrics, validated objective scoring systems for consistent feedback/ Trainer/ training centres accredited	Video 2 external examiner, NOTSS assessment
Porterfield et al Robotic Surgery Education Working Group 2024[39]	Gen. surg Trainees; Universal RAS resident curriculum	Participants; Virtual and in person meetings, written recommendation by members, consensus in day meeting, 100% consensus	8 RAS surgeons/ educators, able to commit	1 consensus meeting/ 100% consensus not specified	Didactic training, simulation (VR, DRY, WET), Video, operative teaching with feedback, bedside assist 10 cases, Gold 20 cases low acuity < 50%, Platinum > 50% mod acuity, Diamond > 50% complex	SimNow: Endowrist manipulation, Camera control, Clutching, Energy Control, 4th arm control, Needle handling, Stapling NTS: “closed loop communication”, safe practices, efficiency	“Pbp”, “modular pathway” feedback, track progress, modern learning management systems, transparent performance /intraoperative metrics/ curriculum director sets learning goals, and tracks progress	Industry equivalency certificate, + letter from program director, Levels: Bronze, Silver, Gold- > technical proficiency; Platinum, Diamond—> procedural proficiency
Pucher et al AUGIS^u^ 2024[50]	Upper GI, Interested surgeons, recommendations for the implementation of robotic upper GI surgery	Participants analysis of discussion and minutes	35 AUGIS members who self-selected by attending the adoption of RAS meeting in 2023 (5 trainees, 19 RAS surgeons, 6 female)	In person meeting, 1 Delphi round among participant /80%	Case observation, didactics, dry- and wet-lab training, followed by cadaveric or animal model operating, prior to proctored cases, surgeons to have min 0.5 full day list/ week during initial learning	Only few skills mentioned: port placement considerations, 4th arm self-assistance, visualisation, competency with interface NTS: Suggest familiarity of the team to improve efficiency and work with each other preferences	Competency-based progression (as judged by proctor or pre-agreed metrics as part of an adoption programme) through AUGIS case complexity^v^ levels 1–3/ proctors min 50 cases, adequate case-mix	Complete approved RAS curriculum + structured proctorship; proctoring to continue until proctor and proctee deem the surgery to be safe, independent, and competent. No set max/min case numbers
Pecoraro et al RAS Kidney transplantation 2024[49]	Kidney transplant surgeon, wishing to gain competence in OKT^w^ and RAKT (min 50 major RAS cases as console surgeon); propose the first structured surgical curricula for both OKT and RAKT	current EAU Guidelines on KT, lit. review, online search of logbook/ curricula for KT worldwide	14/21 RKT experts 1st round, 11/21 2nd round; selected on experience, academic profile and involvement in international associations (EAU, ESTU, ESOT, EKITA, ASTS, ERUS RAKT)^x^	2 Delphi rounds/ ≥ 75% of responders rated 8–10	Presentation, observation, discussion, simulation (3D printed module, porcine model), RAKT case observation, modular, proctored, step-by-step training in OR	Port placement, 10 Specific steps in 3 grades of complexity outlined from entry level to expert NTS: Not defined, expert tips and tricks	Modular training, proficiency-based progression/ host-centre: -2-year activity in RAKT -Dedicated team for transplant -Min volume of 10 RAKT/year	Mentors’ evaluation of proficiency and time to complete each step, final evaluation by a certified independent examiner through a blind process
Harley et al Reconstructive functional Urology 2024[47]	Uro/Gyn surgeons, incl. fellowship surgeons, develop recommendations for credentialing of surgeons performing RFU	Lit. review,	18/35 international experts (14 Urologists, 4 uro-gynaecologists) 14 1st round, 9 2nd round, 13 3rd round	3 Delphi rounds, 80%, Median importance (1–9) and disagreement index given	E-learning/online access to information, Simulation (Dry, Wet Lab), modular training, min 20 cases of mentoring	Competency at positioning, docking, port placement and instrument use in models NTS: Non-technical skills training and operative team training such as decision-making and emergency scenarios	Modular training, proficiency-based progression/certified trainers/proctors, standardised proctor assessment, training centre assessed and audited for volume	Baseline evaluation, 10 bedside observations before training
Huber et al GeRMIQ^y^ 2025[48]	General (Visceral) surgery trainees, find a consensus for the development of a nationwide curriculum for MIS and RAS	Lit. Review	12 national experts, 10 senior physicians, one medical specialist and one junior doctor	2 Delphi rounds, second round at consensus conference, 80% consensus	E-learning, equipment training (through company), Simulation: dry and wet lab, operative training with video assessment	Legal, ethical aspects, patient selection and preparation, trocar placement, docking, pitfalls, two handed movements, camera, dissection, knot tying, suturing, diathermy, use of instruments by assisting staff NTS: Decision making, team training, emergency conversion	Proficiency based progression, collecting points moving through curriculum with existing courses integrated/ DGAV^z^ accredited trainers and training centres, regional structure for access	Feedback and evaluation by a benchmarked evaluation system, Video review by one accredited reviewer, mandatory for specialist exam
Kim et al Society of Thoracic Surgeons 2025[51]	Thoracic surgery trainees, provide a framework for a standardized national robotic curriculum for thoracic surgery trainees	Formulate questions ins meetings and iterative subgroups, lit. review	Unclear, possibly all 19 authors of the paper, expert group ofthoracic surgeon educators and resident representatives interested in robotic education	3 Delphi rounds, 80% consensus	Online modules, Video library, robot components (In-service training), Simulation (VR + yearly wet Lab), case specific milestones	Bedside competency (docking, arm manipulation/ collisions, instrument insertion, troubleshooting, port placement, detailed outline of operative steps NTS: Emergency conversion training once a year	“proficiency based progression, “modular training”, graduated re- responsibility in OR based on milestones, dual console/ provide trained bedside assistant, program ACGME^a1^ accredited	10bedside assists, 5 in thoracic surgery, 90% VR, technical proficiency by objective assessment, yearly video review and analysis by objective evaluators
Wynn et al IMRA/SRS^b1^ 2024[43]	Multi-specialty, including trainees and established surgeons, provide recommendations for objectives, implementation, and certification in robotic training design across diverse training environments internationally	Lit. review, discussion by steering committee, developed by participants as free-text response	24 invited, Response: 1st: 83.3% (20/24) 2nd: 75% (18/24) 3rd: 66.7% (16/24); qualified surgeons, healthcare professionals academics, simulation experts from aviation, curriculum developers and surgical trainees	3 Delphi rounds, 80% consensus, in-person discussion on unclear themes	Online modules, didactic, simulation (VR, laboratory models incl. hydrogel and animal parts), video-based learning, cognitive simulation, intraoperative training	using the 4th arm, control of instruments, depth perception, suturing, cutting, camera control, multi-platform module NTS: NOTSS^c1^ in didactic, simulation and operative training, Awareness of startle effect, Emergency undocking, Managing the surgical team and communication in robotic surgery	Standardised evaluation tool for feedback, proficiency/competency-based progression, stepwise multimodal modular approach, basic to advanced general to specialty specific modules, relevant performance metrics/benchmarks in real surgery/ proctor with adequate training to teach	Minimum number of supervised cases (number not specified), number of cases to reach competency may vary, sign off based on competency/ proficiency. Video review (not specified if this is to be external/ blinded)

Qualitative studies	Target/aim	Generation of themes/method of consensus	No/type of participants	Methods/% of consensus	Curricular components	Skills required technical non-technical (NTS)	Educational theory and requirement	Assessment and certification
Dulan et al. 2012 Texas South West [31]	All specialties all surgeons; Qualitative, pre-proc. curriculum	Participants, 10 RAS cases skills deconstruct. Qualitative, -interviews -10 cases observed/ skill deconstruction	6 RAS surgeons (min 10 cases)	Interviews with experts	E-learning, half-day interactive session, simulation (DRY), 9 inanimate task developed assessing 23 skills	Console settings, docking, trocar triangulation, robotic positioning (arm positioning), communication, Energy sources, robot component names, camera, clutching, instrument names/ exchange, 4th arm control, wrist articulation, hand–eye coordination, atraumatic tissue handling, dissection (blunt/fine), suturing, cutting, retraction NTS: Closed loop communication	“Pbp”, deliberate practice, proficiency-based metrics/	Assessment on dry lab skills, port placement
Green et al. 2019[21]	Gen. surg Trainees, Qualitative, improve RAS introduction into apprentice-based training	Participants, Qualitative interviews	24 RAS surgeons with OR resident contact	Qualitative Interviews at a SAGES conference	Simulation (VR, DRY), operative practice, DUAL console, increased access, teach differences to laparoscopy	Tool vs. technique: familiarise learner with robotic tool outside the OR docking, e.g. Instrument exchange, clutching, moving between arms at console Other skills not mentioned NTS: No mention	“Modular” training, dual console	No mention
Zhao et al 2020[34, 35]	All specialty, Surgical trainees, Qualitative, barriers and perception towards RAS training	Participants, Qualitative interviews	20 residents, 7 attendings in Gen surg	Semi-structured interviews	Simulation (VR, DRY, WET), Feedback, bedside assist, operative training, improved access, dual console	No mention NTS: No mention	“Modular approach”, Pbp, Feedback/ Training centres: -Adequate case volume, -training infrastructure, -educational culture	No mention
Jogerst et al 2023[40]	Gen. surg Trainees; RAS training recommendations for curriculum and assessment	Participants, Qualitative interviews	34 High volume RAS (> 100 cases/yr) surgeons/ educators academic an community general surgery urology gynaecology	Semi-structured interviews	E- learning, simulation, bedside teaching, intra-op teaching, adequate case volumes	Not defined NTS: No mention	Pbp, benchmark progress tracking/ adequate case volume, completed faculty learning curve, resident access to training resources (Sim, courses, tissue models)	Video review

^a^VR Virtual Reality

^b^DRY dry lab

^c^FRS Fundamentals of Robotic Surgery

^d^Pbp = Proficiency based progression, “Pbp” = exact words are not used in study, but described concept matches the idea, for easier comparison these words are used in the table

^e^ERUS ERUS European Association of Urology (EAU) Robotic Urology Section

^f^WET wet lab

^g^OR Operating room

^h^RARP Robotic assisted radical prostatectomy

^i^Modular training = segments of complex tasks are taught in logical series, “modular training” = exact words are not used in study, but described concept matches the idea, for easier comparison

^j^ESTS European Society of Thoracic Surgeons , EACTS European Association for Cardio-Thoracic Surgery

^k^SERGS Society of European Gynaecologic surgery

^l^RAPN Robotic assisted partial nephrectomy

^m^ORSI OLV(Onze Lieve Vrouwziekenhuis) Robotic Surgery Institute, OCERT ORSI Consensus Meeting on European Robotic Training

^n^BIARGS British and Irish Association of Robotic Gynaecological Surgeons

^o^HPB Hepatobiliary surgery

^p^FTRS Fundamentals of Thoracic Robotic Surgery

^q^PGY Postgraduate year

^r^RAMIE Robotic assisted minimally invasive esophagectomy

^s^ASiT Association of Surgeons in Training

^t^CCT Certificate of Completion of Training

^u^AUGIS Association of Upper Gastro Intestinal Surgery Great Britain and Ireland

^v^AUGIS case complexity: Level 1 cases include cholecystectomy, primary anti-reflux surgery, primary hernia, Level 2 cases include paraesophageal hernias, subtotal gastrectomy, Level 3 cases include esophagectomy

^w^OKT Open Kidney Transplantation , RAKT Robotic Kidney Transplantation

^x^European Association of Urology [EAU],Section of Transplantation Urology [ESTU], the European Society of Organ Transplantation [ESOT], the European Kidney Transplant Association [EKITA], the American Society of Transplant Surgeons [ASTS] and ERUS RAKT Working Group

^y^GerMiQ National Curriculum for Minimally Invasive and Robot-assisted Surgery in Germany

^z^DGAV- Deutsche Gesellschaft Allgemein und Viszeralchirurgie/German Society for General and Visceral Surgery

^a1^ACGME Accreditation Council for Graduate medical Education

^b1^IMRA/SRS International Medical Robotics Academy/ Society of Robotic Surgery

^c1^NOTSS Non-technical skills for surgeons

We identified several recurrent themes mentioned in the analysed publications, which can be grouped according to Kern’s curricula framework.

*Step 1*: Problem identification and general needs assessment: curriculum standardisation.

Most publications reported a high level of agreement on the need for a standardised curriculum [19, 22, 24–27, 29, 36, 40–43, 47–52], or included this as their aim [[31, 32, 37, 39].

Members of the first European Association of Urology Robotic Urology Section (ERUS) curriculum consensus study remarked that training was “random”, and the “learning curve” a “risk for patient safety” and no longer acceptable, therefore RAS should be standardised globally with “national variations” [25].

In a qualitative interview study by Jogerst et al. all specialties agreed on pooling resources’ to prevent ‘reinventing the wheel across subspecialty silos’ [40].

*Step 2*: targeted needs assessment: Two learning curves and the needs of surgical trainees.

The requirement for a tailored curriculum after a baseline skills assessment was mentioned in multiple publications.[19, 22, 24, 25, 42, 43, 47]The needs of trainees, who require training robotic as well as procedural skills were emphasised [19, 39, 40]. Studies mentioned the importance of and difficulty with trainee access to RAS training [38, 43]. A risk of trainees taking on “observer roles” and just “checking off” operation was highlighted [23]. Access to console training was identified as a specific challenge[21, 23, 34, 35, 40], with the dual console as a facilitating factor [20, 21, 23, 25, 26, 34, 35, 51].

Pucher et al. specified “*in units with an established robotic programme, trainees should have access to, and training with, robotic platforms*”.[50]Studies disagreed whether experienced surgeons also had to go through a procedure specific curriculum, or if a basic RAS skills curriculum would suffice [22, 29, 41].

*Step 3*: Goals and Objectives: Common skills required and educational content.

A significant number of studies identified specific RAS psychomotor skills that are required to achieve competency. These studies had considerable agreement on the skills needed. Specific technical skills, such as port placement and RAS arm ergonomics, control of the robot and tissue handling were identified by Dulan et al. and appear to be reiterated by many consensus studies thereafter. [20, 22, 24–26, 28, 30–32, 37, 38] (see Table 3) The British and Irish Association of Robotic Gynaecological Surgeons (BIARGS) consensus curriculum consists of a list of skills needed to be demonstrated by trainees, without providing a teaching syllabus. recommendation) [52].

**Table 3:** Summary table of skills content

Knowledge	Psychomotor skills/ ‘Buttonology’	Operative Skills Technical	Operative Skills Non-Technical
Patient selection (8/28), pre- and post-operative considerations, Theatre team efficiencies (6/28), knowledge of instruments	Bedside: Patient positioning, laparoscopic skills troubleshooting (9/28), optimal **port placement for triangulation** (21/28), burping, **docking** (18/28) (set-up and draping of the robot), **robotic arm** (14/28)-ergonomics (clashes), Console: **camera control** (17/28), safe **tissue dissection** (17/28) (sharp and blunt) and **tissue handling** (14/28) (no tactile- feedback awareness off-screen), knot tying, **suturing** (16/27) (interrupted and continuous), transfer/ two-hand coordination, correct wrist articulation (9/27), manoeuvring the robotic arms, using the **energy sources** (diathermy) (14/28)	Procedure/ specialty specific Definition by specialty society: Operative/ Modules steps defined (6/28) e.g Basic colorectal: vessel dissection, bowel resection, intracorporeal anastomosis, pelvic dissection	“Closed loop” **communication** (19/28) **decision making** (15/28), **emergency scenarios/ undocking** (20/28), **situation awareness** startle effect (13/28), leadership (9/28)

In brackets the number of studies mentioning word/ concept as analysed with NVivo

Bold > 50% studies mention skill

*Step 4*: Educational strategies: Common curricular components and educational strategies.

E-learning and video libraries were the most common modalities used for knowledge training. Simulation is included in psychomotor skills training in all studies. This includes VR simulation, dry lab, wet lab (animal or cadaver) and team training simulations. Dry lab and VR simulation were mentioned most. The cadaveric lab was mentioned in seven studies, voted on as without benefit once[29] and mentioned to be replaceable by models twice [43, 47]. The European Society of Coloproctology (ESCP) guideline working group upgraded the recommendation for simulation training to a *strong recommendation* even though they stated there was a low level of evidence in the RAS colorectal literature [52].(See Table 1 and 2).

In the recent IMRA/SRS consensus study only 11% of experts agreed that the use of VR simulators would provide enough simulation training [43].

Operative training is mentioned in publications concerning comprehensive and procedure specific curricula, with the most common specification being for training to be ‘modular’ and ‘proficiency based’. Telemonitoring, was included as a ‘viable option’ in two specialty specific curricula [[24, 25] and the ESCP guideline [52].

Non-Technical skills (NTS) training was commonly, but not unanimously, agreed on. Two specialty specific general surgery publications reported a lack of consensus on NTS training. [22, 24] Common aspects of NTS that were suggested as being important included team-training, emergency undocking and communication training. Several publications suggested video review as a teaching tool for RAS training. [[19, 22, 29, 36, 39, 43, 48, 51] (Table 4).

**Table 4:** Summary table of curricular components

Knowledge	Psychomotor training	Operative training	Educational concept	Assessment	Requirements
**E- learning** 20/28 Video library 6/28	**Simulation** (28/28) **VR**(21/28), **Dry lab** (19/28), **Wet lab**17/28/ cadaver (7/28)	**Modular training** 16/23*, Video review 8/28 **Team training** 15/28 **NOTS skills** 18/28	**Proficiency /competency-based progression** 25/28: pre-defined **benchmark/ metrics/** error modes18/28, Feedback 9/28	Anonymous, **external video** 14/28, continued outcome reporting 8/28 certification by institution 3/28 society 1/28	**Specifically trained robotic educators** (17/28), **training centres must meet specific requirements** e.g. Volume (13/28), independence from industry (12/28)

^*^Studies not commenting on operative training excluded[31–33, 36, 38]; Bold > 50% of studies mention the component

Deliberate practice as an educational concept of psychomotor learning was mentioned in several publications [23, 30, 31, 33, 36]. (Definitions of educational concepts in Table 5).Additional publications highlighted the importance of feedback [20, 22, 24, 38, 39, 41]. Proficiency based progression (PBP), or a description of the concept, (competency-based progression) was included in almost all the studies examined. Most studies did not define competency or proficiency, and the terms appeared to be used interchangeably. [43]

**Table 5:** Definitions of educational concepts

Educational concept	Definition
Deliberate Practice	Activity to improve performance, requiring: ∙A task with a well-defined goal, ∙Motivation to improve, ∙Feedback ∙Ample opportunities for repetition/refinements [53]
Proficiency based progression	Surgical training methodology, ∙Identification of objective performance metrics (optimal/suboptimal) for operative procedure ∙Training until pre-defined benchmarked proficiency level is met ∙Continuous formative feedback during practice ∙Proficiency level is based on mean performance of experts/experienced practitioners[54]
Modular training	Educational concept of breaking down a complex learning task into separate parts which can then be mastered in a stepwise fashion with rising complexity until the entire task is performed [55]. As opposed to completing a task in chronological order

The change to curricular philosophy is emphasised by Vanlander et al.: “Traditionally, case volume and exposure created expert surgeons, whereas nowadays metric-based deliberate practice determines improvement and may create experts” [33].

‘Modular training’ was the most common educational concept used to structure the intraoperative curriculum. [20–22, 24–27, 29, 30, 33–35, 39, 41, 43, 47, 49, 51, 52]

Harley et all reported modular training to be the ‘cornerstone of contemporary surgical education’ in the phase of applying learned knowledge and skills to live surgical procedures. ‘Complex operative procedures’ were deconstructed into their ‘fundamental steps’. These operative components were learned by the trainee along increasing levels of difficulty in the transition to independent practice under mentor supervision [47].

Step 5: Requirements for Implementation: Surgical teachers, training centres and the involvement of Industry.

Jogerst et al. suggested that the ‘culture of teaching’, and the ‘culture of being willing or unwilling’ are the main factors determining whether the implementation of a training curriculum for RAS will be successful[40].

Multiple studies mentioned surgical trainer or training centre requirements[22, 24, 36, 38, 46–48, 52], 44] Herron et al. mention unbiased, confidential mentoring. [19] Multiple studies reported on the necessity for adequate centre case volume[22, 24, 26, 27, 29, 34, 35, 38, 40, 41, 47, 49], teaching infrastructure[21, 29, 30, 35] and specific requirements for accreditation of surgical teachers [19, 20, 22, 24, 27, 29, 33, 36, 38, 40, 41, 43, 47, 48, 50]. train-the trainer course [43, 52].

The IMRA/SRS consensus study mentioned component training for multilevel learners as a solution for improving access for multiple trainees [48].

Ownership of responsibility for training provision and accreditation was a recurrent theme in the studies in this review. Involvement of industry in robotic training was mentioned multiple times, with studies expressing a need for RAS teaching to be independent from industry [20, 36, 51], and adherent to standards defined by surgical societies [22, 24, 25, 42, 48, 50]. Accreditation of training was identified as the responsibility of the surgeon’s institution[19, 20, 36, 50]. Veronesi et al. demanded that “*every effort should be made to prevent the regulation of training activities and accreditation by manufacturers alone*” [29].

Stefanidis et al. referred to the 2009 SAGES definition of a proctor (assessing the ability to operate) and preceptor (teaching)[56] and reached a strong consensus in favour of the proctor being independent, not selected by industry. Preceptor and proctor are advised to be able to take part in the operation, if necessary, for patient-safety reasons [36]. Pucher et al. found a “*potential of commercial and political bias*” as well as risk of the current practice of proctor selection being *“by industry alone without specialty society input and oversight”*.[50]

Wynn et al. emphasised collaboration with industry regarding standardisation of design and verification of basic skills training as well as for data collaboration and research on the recognition of proficiency [43].

*Step 6*: Assessment and Evaluation.

‘Benchmarked’ ‘metrics’ were commonly suggested as a requirement for progress tracking and assessment [19, 22, 24, 27, 29–33, 36, 38–40, 43]. Multiple papers described anonymous video reviews of full-length index-cases by two blinded examiners before independent practice was permitted [22, 24–27, 29, 36, 38, 40, 42, 43, 47, 48, 51, 52] Additional case logs were required in one study [24]. Only gynaecological consensus studies specified curriculum case volume (10–20 cases) [28, 41, 47]. Veronesi et al. mention the requirement for three proctored cases[29]. Other consensus documents mentioned adequate volume[19, 40] and ‘benchmarked proficiency’ instead of case volume [22, 24]. Institutional certification was included in several studies [19, 20, 36, 50]. Pucher et al. advised that independent operating was only possible once the “*proctor and proctee”* were both satisfied with “*safety, independence and competence*”. The responsibility for” *clinical competence and patient outcomes*” lies with the surgeons and the “*institutions governance policies*” [50].

## Discussion

The aim of this study was to synthesise expert opinion on the ideal content and structure of a RAS training curriculum. The main findings were that there was agreement on curriculum standardisation and its components, educational structure, and on the specific psychomotor skills needed to perform RAS. The curriculum should be adapted to a surgeons' skill level, be led by well-trained trainers utilizing benchmarked assessments and delivered in centres with adequate robotic case volume.

Expert consensus highlights that e-learning and simulation are essential curricular components for RAS training. Well-constructed e-learning modules have been shown to be beneficial in delivering cognitive teaching in surgery [57]. For example, a recent randomised controlled trial from the ORSI academy (OLV[Onze Lieve Vrouwziekenhuis] Robotic Surgery Institute’ utilised by the ERUS curriculum) supported the superiority of their proficiency- based- progression (PBP) e-learning over traditional lectures and self- study [58]. E- learning is scalable, convenient to the learner and can be delivered at minimal cost, yet many RAS trainees rely solely on industry-provided e- learning content [59]. The establishment of a video library, recommended in multiple consensus studies, has been shown to increase trainee participation in RAS [60].

Consensus studies have unanimously supported the use of simulation training before live operating is attempted. A variety of simulation modalities, such as VR, dry and wet lab, have proven effective in skill acquisition and transfer of those skills to clinical practice. [61, 62] VR simulation is cited slightly more frequently compared to dry and wet lab modalities, (see Table 2) however, 89% of RAS experts in the IMRA/SRS consensus study disagreed that VR simulation training alone is sufficient for a RAS curriculum [43]. VR simulation allows the novice to learn using the machine, but the subtleties of the visual cues necessary for operating are not adequately conveyed. Furthermore, VR simulator scores do not reliably distinguish between advanced novice and expert surgeons [63], Dry lab and wet lab simulation has demonstrated benefits over VR training [62], although the wet lab has not been as extensively examined in RAS compared to other simulation modalities. 3D printed models, appear to be a promising alternative for animal or cadaveric training [64]. These findings highlight the value of an approach integrating e-learning with a wide range of simulation modalities.

Consensus studies have identified a range of RAS skills, and these have been incorporated into simulator tasks and preclinical curricula. Dulan et al. deconstructed RAS skills in their qualitative study and developed an inanimate simulation curriculum including these skills. They established expert benchmarks and face, content and construct validity for the inanimate simulation curriculum [31, 65, 66]. Later the University of Texas South West published a VR evolution of the skills curriculum [67]. The FSRS and the FRS curriculum also involved preclinical, inanimate and VR simulation for RAS psychomotor skills [32, 68–70]. Simulation training was shown to improve simulator scores, without improvement in the ergonomic habits of novices [71]. More recently, RAS ergonomic skills, such as awareness of robotic arm position, docking and avoidance of clashes have been addressed with specific teaching sessions [72, 73].

Modern educational theory was included in many publications with the mention of feedback, benchmarks and proficiency-based progression training. Ericsson et al., deconstructing ‘innate talent’, discovered that ‘deliberate practice’ is necessary for optimal psychomotor learning. During deliberate practice a motivated learner practises a significant task with a clearly defined goal and receives feedback for improvement [74]. Proficiency-based progression training, first introduced by Satava et al. in 1993, builds on deliberate practice. Training occurs to predefined benchmarks of proficiency after which the next step in training can be attempted [54].

RAS experts, in the studies that were reviewed, include these educational concepts in simulation and intraoperative training. Simulation provides the ‘ample opportunities for repetition’, [74] necessary for deliberate practice. VR simulation enables automatic feedback and assessment with simulator metrics. However, VR simulators do not offer more than basic advice on how to improve performance [35]. Trainees who receive teaching, while practising the FRS curriculum, outdo their peers using simulator guidance alone [75]. Innovative models for self-teaching on the simulator have been examined but have not been not implemented into RAS curricula [76, 77]. Multiple consensus studies agree on modular or component-based intraoperative training, by increasing the complexity of operative steps. Operative modules for index cases have been predefined by several specialty specific consensus studies [26, 27, 78]. This outlines a clear training path instead of “*doing the whole case at once*”, which may result in minimal involvement of the trainee and the possibility of just having a case “*signed off*”. [23] Training by using modules of increasing complexity may encourage deliberate practice to reach predefined benchmarks. Modular training, as outlined by many of the consensus studies, with objective feedback to proficiency, will transport optimal psychomotor training into the operating room.

The teaching skills of mentors and the use of unbiased feedback with the aid of scoring systems or video review as a teaching tool, are highlighted as important aspects of intraoperative training. Current evidence suggests that teaching delivery could be improved, even in centres with established RAS training pathways [79]. RAS may require unique intraoperative mentoring skills [80]. RAS-specific aspects of providing contextual explanation for trainees, optimizing autonomy and the multimodal use of RAS teaching tools have been identified as important aspects in RAS mentoring [79]. RAS train-the-trainer curricula have been developed[10, 81] but have not been widely implemented. Several of the studies proposed accreditation of RAS surgical trainers and training centres by volume, operative experience and ability to teach. Accreditation could be done by surgical societies.

Most studies required the achievement of benchmarked proficiency for curriculum completion, instead of volume of cases completed. For comparable assessment of RAS operative skills, validated scoring tools, such as GEARS (Global Evaluative Assessment of Robotic Skills) or ROSAT(Robotic operative structured assessment tool), have been proposed.[82]Automated performance metrics, computing surgical skill from instrument motion tracking, event data and surgical videos are increasingly being developed and in future may provide valuable information for training and assessment [82].

During RAS training on the dual console, the percentage of active involvement can be easily and reliably documented through the metric, ACT (Active Control Time), and this can be used as a surrogate marker for successful teaching [83]. A significant increase in trainee ACT (intraoperative entrustment) was seen after the introduction of RAS training interventions [60]. The surgical robot facilitates recording of intraoperative videos. Video is already in use in robotic training but underutilised in current European surgical training curricula [14, 60].

Several consensus studies agreed on the need for unbiased external video review by more than one examiner using a scoring system before independent operating. To our knowledge there are no studies available comparing the outcome of RAS surgeons who have had objective video assessments, to their peers who received proctoring only.

Multiple consensus studies recommended non-technical skills (NTS) for RAS be included in a curriculum. Situational awareness, communication and emergency team training were the most mentioned. Even though NTS does not receive the same interest as technical skills[84], the importance of NTS for team functioning and the safety of patients is gaining wider acceptance in the surgical community.[85]. By removing the surgeon from the bedside, RAS places an increased demand on surgical NTS such as situational awareness and communication.[86, 87]. An emergency scenario is a rare event and conversion skills cannot be learned during routine bedside assisting. Dedicated team simulation scenarios have shown a dramatic improvement in time to undocking, and team coordination [88]. Awareness and practice in RAS NTS will prevent skills gaps that may have a detrimental impact on the patient.

The uptake of RAS curricula in the USA is more advanced than in Europe [89]. Since the ACGME guidance from 2022 [90] multiple programs have started to introduce robotic surgical training for their trainees. However, even in the USA, considerable variation in programs remains and no standardised curriculum or curriculum structure has been introduced to date [91].

Most robotic training in Europe is currently provided by industry.[14]Even though several independent societal curricula have been designed, validated and implemented (e.g. from ERUS[92–94] and ESCP) (e.g. ORSI[95] few surgeons receive optimal robotic training[96–98]. Proctors are engaged by industry, their accountability is not openly published, and the division of teacher/ assessor not clearly drawn [99]. Whilst many surgeons have benefitted from industry sponsored training, the surgical workforce of tomorrow is the responsibility of the national bodies who control surgical training e.g. the surgical colleges, statutory education bodies and specialty associations. The goal, therefore, should be to find the optimal training pathway for robotic surgeons based on the best evidence and independent from industry.

In a time when the surgical robot is not available at every training site it is difficult to mandate completion of RAS training for all surgical trainees. We can, however, easily mandate training for surgical trainees who are already training in sites with a surgical robot, ensuring trainee participation and adequate training in regions where RAS is an important part of service delivery. As surgical educators, we can and must agree on a set of requirements to achieve independence in RAS operating. We can specify a standard of independence and skill which, in contrast to traditional surgery, is easily measurable on the RAS console. Surgeons could be required, by surgical colleges, to complete an advanced specialty specific RAS curriculum or have been video assessed, on their index procedures. For improved access to robotic training for trainees, educating bodies should mandate that a standardised RAS surgical training pathway is made available in organisations offering robotic surgery. To improve the evidence that supports specific structured RAS curricula, we need to evaluate the available curricula by investigating measurable metrics of skill acquisition and patient outcomes, comparing surgeons who have undertaken specific curricula with surgeons who have received the current unstructured training. This is step 6 in Kern’s curricular development model and re-informs the curricular improvement cycle. The main curricula requirements have been summarised in Table 6.

**Table 6:** Summary of RAS expert opinion of necessary curricula content and structure

Skills	Curricula content	Implementation requirements	Curricula structure
Port placement Docking Robotic arm ergonomics Camera control Tissue dissection Tissue handling Suturing Energy sources	E-learning	Online	Proficiency/competency based progression to pre-defined benchmark metrics
	Simulation (VR, Dry lab, wet lab)	In-house and/or provided as course	
	Modular intraoperative training	Specifically trained RAS educators	
		Accredited training centres with adequate volume	
Procedural skills			
RAS communication Decision making Situation awareness	NOTS training in Simulation and Modular intra-operative training		
Emergency conversion scenarios	Team training/simulation		
Curriculum completion	Objective unbiased (external) assessment e.g. video review		

## Strength and limitations

This is a novel systematic review of expert opinion on the best content for RAS curricula. We have combined evidence over a large time span and have included multiple specialties. This, therefore, is the largest systematic review of its type. Wynn et al. conducted a review of RAS consensus papers, before embarking on their own Delphi project, but included fewer studies (n = 5 compared with n = 28) [43]. Due to a lack of good evidence, multiple curriculum developers have turned to expert opinion to develop their content and structure of RAS curricula. Here we present a summary of a broad range of expert opinion, outlining commonality and differences.

The main limitation of this review is the low quality of evidence from expert opinion. In addition, there is considerable overlap in authorship and sponsorship among studies. This may have been due to the Delphi process and a concentration of expertise in RAS education, and of the involvement of specialty societies, in a particular international community of practice[19, 22, 24–27, 29, 30, 32, 33, 36, 38, 41, 43]. Medtronic, Minneapolis MN, USA supported multiple authors and multiple consensus studies, with four studies specifically thanking the same Medtronic company representative [22, 24, 29, 46]. Intuitive Surgical, Sunnyvale CA, USA funded several authors [25, 26], and provided funding for one study [39]. Intuitive Surgical, Sunnyvale, CA USA has, for many years, been the only manufacturer of the surgical robot and has played a major role in robotic training.

Another potential limitation could be the positionality of the lead author as a surgeon with experience of surgical- and robotic training in two different European Healthcare systems. Theoretically this could influence or bias the interpretation of the data. However, this was mitigated by including a wide range of experience in the author research group and co-authors: surgeons in robotic training, trainee surgeons, a surgical and a non-surgical educationalist and an academic non-robotic surgeon.

Future research presenting participants skills and patient specific outcome data is necessary to avoid sole reliance on expert opinion on curricula content. The assessment of trainees after curriculum completion is an important part of Kern’s curricular model, which not only accredits the skills gained but also feeds back towards curriculum evolution. [17]Comparable, objective participant skills assessments, which include patient specific data, will provide program developers with more robust evidence, when costly educational resources are allocated during RAS curriculum implementation.

## Conclusion

The consensus on RAS training proposes a framework supported by today’s understanding of optimal psychomotor learning. The suggested assessment and credentialing process attempts to improve patient safety and reduce bias. To improve RAS training for surgeons and trainees surgical colleges and associations must agree on a platform agnostic common RAS curriculum that incorporates simulation training. The learning outcomes and proficiencies gained must be benchmarked using assessment metrics which are specific to both specialities and procedures. RAS training should be delivered in modules by centres who have credentialled trainers and an adequate volume of robotic surgery. Proficiency should be achieved before independent operating is permitted.

## Supplementary Information

Below is the link to the electronic supplementary material.

Supplementary file1 (DOCX 270 KB)

Supplementary file2 (PDF 136 KB)

## Data Availability

No datasets were generated or analysed during the current study.
