# Correction to: Expert consensus on best content of a robotic surgical curriculum: a systematic review

**DOI:** 10.1007/s11701-026-03184-0

**Published:** 2026-02-16

**Authors:** Anna K. Kieslich, Ruari Jardine, Hussain Ibrahim, Areeg Calvert, Kenneth G. Walker, Kim A. Walker, Angus J. M. Watson

**Affiliations:** 1 NHS Highland, Raigmore Hospital, Old Perth Road, Inverness, Scotland IV2 3UJ UK; 2 Centre for Healthcare Education Research and Innovation (CHERI), University of Aberdeen, Aberdeen, Scotland UK

In the original version of this article, abbreviations in front of the explanatory text in the footnote j and w were incorrectly mentioned as superscript.

Footnote j and w, which previously read as

^j ESTS^European Society of Thoracic Surgeons, EACTS European Association for Cardio-Thoracic Surgery

^w OKT^ Open Kidney Transplantation, RAKT Robotic Kidney Transplantation

but should have read.

^j^ ESTSEuropean Society of Thoracic Surgeons, EACTS European Association for Cardio-Thoracic Surgery.

^w^ OKT Open Kidney Transplantation, RAKT Robotic Kidney Transplantation.

The original version of the article has been corrected.

