# Supplementary material for: Expert consensus on best content of a robotic surgical curriculum: a systematic review
Source: J Robot Surg. 2025 Oct 28;19(1):721. doi: 10.1007/s11701-025-02893-2 (PMC12559105; doi:10.1007/s11701-025-02893-2)
Supplement: Supplementary file 2 — Supplementary file2 (PDF 136 KB) [file 11701_2025_2893_MOESM2_ESM.pdf]

Search Strategy: Essential components of a robotic surgical curriculum for surgical trainees

2024/01/18 Medline:

1.exp Robotic Surgical Procedures/2.robot assisted surgery.tw,kw.

3.(robot$ adj2 surger$).tw,kw.

4.1 or 2 or 3

5.exp Education, Medical/6.exp Curriculum/ *7.exp Motor Skills/* *(taken out 17 jan2024)*

8.exp Simulation Training/9.exp Clinical Competence/10.exp "Internship and Residency"/

11.exp Learning Curve/ 12.training.tw,kw. 13.skill$.tw,kw. 14.(surgeon? adj3 training).tw.

15.(surg$ adj3 training).tw,kw.

16.5 or 6 or 7 or 8 or 9 or 10 or 11 or 12 or 13 or 14 or 15

17.4 and 16 18.limit 17 to yr="1997 -Current"

Embase 2024/01/018:

1.exp robot assisted surgery/

2.exp medical education/3.exp high fidelity simulation training/

4.exp simulation training/5.exp surgical training/6.exp curriculum/7.exp curriculum development/

8.exp clinical competence/9.exp learning curve/10.exp skill/11. exp skill retention/

12.(robot$ adj2 surger$).tw,kw.13.(surgeon$ adj3 training).tw,kw.14.training.tw,kw.

15.exp training/16.skill$.tw,kw.

17.2 or 3 or 4 or 5 or 6 or 7 or 8 or 9 or 10 or 11 or 13 or 14 or 15 or 16

18.robot assisted surgery.tw,kw. 19.(robot$ adj2 surger$).tw,kw. 20.1 or 18 or 19

21.17 and 20 22.limit 21 to yr="1997 -Current"

Scopus 20240116:

((TITLE-ABS-KEY ("robot* surg*")) OR (TITLE-ABS-KEY ("Robotic Surgical Procedures")) OR (TITLE-ABS-KEY ("robot* assisted surg*"))) AND ((TITLE-ABS-KEY ("surg* train*")) OR (TITLE-ABS-KEY ("curriculum*")) OR (TITLE-ABS-KEY ("clinical competence")) OR (TITLE-ABS-KEY ("internship and residency")) OR (TITLE-ABS-KEY ("simulation training")) OR (TITLE-ABS-KEY ("learning curve"))) AND PUBYEAR > 1996 AND PUBYEAR < 2025

Psych Info 20240116:

exp Computer Assisted Surgery/ OR robotic surgery.mp. OR

robot assisted surgery.mp.

AND

exp Curriculum/OR

exp Medical Education/OR

exp Clinical Methods Training/ OR exp Postgraduate Training/ OR exp Training/

exp Competence/ OR exp Simulation/

exp Clinical Practice/

Cinahl 20240118 :

S17:S15 AND S16

S16:S4 OR S5 OR

S6 OR S7 OR S8 OR S9 OR S10 OR S11 OR S12 OR S13 OR S14

S15:S1 OR S2 OR S3 S14:"learning curve" S13"surgical training"

S12"training" S11(MH "Education, Medical, Continuing")

S10(MH "Education, Medical+") S9(MH "Curriculum Development")

S8(MH "Curriculum+") S7(MH "Clinical Competence+")

S6(MH "Psychomotor Performance+") S5(MH "Motor Skills+") S4(MH "Skill Retention")

S3robotic surgery S2robot* assisted surgery S1(MH "Robotic Surgical Procedures")

PubMed 20240116

("education, medical"[MeSH Terms] OR "medical education"[Text Word] OR "education, medical, continuing"[MeSH Terms] OR ("Curriculum"[MeSH Terms] OR "Curriculum"[Text Word]) OR ("simulation training"[MeSH Terms] OR "simulation training"[Text Word]) OR ("clinical competence"[MeSH Terms] OR "clinical competence"[Text Word]) OR ("internship and residency"[MeSH Terms] OR "internship and residency"[Text Word])) AND ("robotic surgical procedures"[MeSH Terms] OR ("robot*"[All Fields] AND "surg*"[All Fields] AND "procedur*"[All Fields]) OR "robotic surgical procedures"[All Fields] OR ("robot*"[All Fields] AND "assist*"[All Fields] AND "surg*"[All Fields]) OR "robot assisted surgery"[All Fields])
